# Predictions and Experiments on the Distortion of the 20Cr2Ni4A C-ring during Carburizing and Quenching Process

**DOI:** 10.3390/ma15124345

**Published:** 2022-06-20

**Authors:** Yongming Yan, Yanjun Xue, Wenchao Yu, Ke Liu, Maoqiu Wang, Xinming Wang, Liuqing Ni

**Affiliations:** 1 Central Iron & Steel Research Institute Company Limited, Beijing 100081, China; 15110562925@163.com (Y.X.); yuwemchao@nercast.com (W.Y.); maoqiuwang@hotmail.com (M.W.); 2 Jianglu Machinery and Electronics Group Co., Ltd., Xiangtan 411100, China; liuke820x@126.com; 3 School of Material Science and Engineering, Xiangtan University, Xiangtan 411105, China; wangxm@xtu.edu.cn (X.W.); 202121551539@smail.xtu.edu.cn (L.N.)

**Keywords:** martensite transformation, carburizing and quenching process, simulation, distortion

## Abstract

This paper focuses on the effect of gear steel on distortion due to phase transformation in carburizing and quenching. The carburizing and quenching process of C-rings under suspension was studied by using the finite element method based on the thermo-mechanical theory, considering phase transformation. The phase transformation kinetics parameters, depending on different carbon contents, were measured by Gleeble-3500. The distortion behavior of the carburized C-ring during the cooling stage was analyzed, as well as the carbon concentration distribution and martensite volume fractions. The accuracy of the simulation was also verified by comparing the experimental data with the simulated result of the distortion and microstructure. A reliable basis is provided for predicting the distortion mechanism of gear steels in carburizing and quenching.

## 1. Introduction

Carburizing and quenching are often used to improve the mechanical properties and the service life of a gear [1,2]. However, the deformation caused by carburizing and quenching leads to dimensional deviation and structural instability of the gear, increasing the machining workload and reducing the production efficiency. Controlling deformation has always been a difficult problem in the manufacturing of gear parts, especially for thin-walled parts. There are many factors that affect distortion during carburizing and quenching, such as the carburizing and quenching temperature, quenching medium, material composition, size of the gear, etc. [3,4]. Ingredient differences between the surface and core after carburizing make the stress–strain derivation law during the quenching process become very complicated. With the development of computer simulation technology, theoretical models and finite element analysis methods have gradually improved, providing new ways to control heat treatment distortion. Some scholars have used numerical simulation to conduct in-depth analysis of carburizing and quenching [5,6,7,8,9,10]. Silva et al. simulated the quenching process of an AISI 4140 steel C-ring in oil, covering the analysis of the distortion caused by both thermal contraction and phase transformation [6]. Farivar et al. [9] investigated the effects of a modified hardening temperature and different soaking times on the core microstructure, the final dimensional stability, and the mechanical properties of 20MoCr4 steel.

Only with correct and reliable material parameters can reliable numerical simulation results be obtained. However, there are many problems in the simulation of material parameters at present, such as inconsistent data grades, imperfect material data, or even no data. Among them, the K-M formula is often used for the calculation model of martensite transformation [11,12], and the parameter α in the formula is generally selected as 0.011, which is obviously not rigorous enough. The error of martensite transition temperature calculated by the empirical formula is large. Different materials should be calculated by corresponding experimental measurements. The method was developed to obtain the phase transformation expansion and transformation plasticity of steel during phase transformation and heat treatment, and the coefficients of phase transformation expansion and transformation plasticity can be calculated based on the kinetics theory of phase transformation [13,14,15]. The kinetics of the martensitic transformation in three carbon steels (C60, C70 and C80) have been studied using the acoustic emission (AE) technique during continuous cooling in the Gleeble 1500 by van Bohemen [16]. Therefore, in order to improve the accuracy of the simulation of the carburizing and quenching process, this study firstly conducted thermal expansion experiments on smelted materials with different carbon contents to obtain the martensitic transformation parameters. Then, the finite element method was used to predict the properties and distortion of the C-ring, suggesting optimization of the carburizing and quenching process.

## 2. Numerical Simulation Theory of Carburizing and Quenching

**Figure 1:**
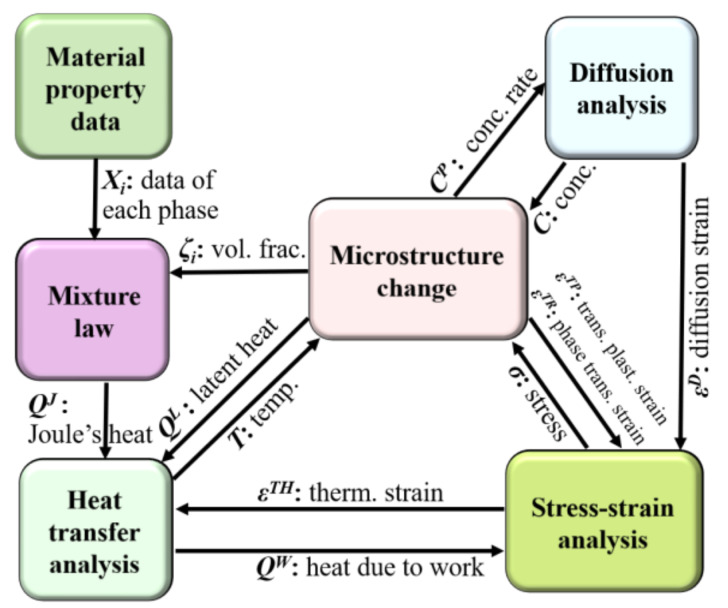
Structure of the heat treatment simulation system and flow of data.

The carburizing and quenching process involves a complex continuous medium thermodynamic theory and requires consideration of the coupling between the carbon concentration diffusion field, temperature field, phase transformation kinetics, and tissue distribution, as well as the inelastic stress–strain field [10]. A concrete structure of the heat treatment simulation system is shown in Figure 1. Firstly, the diffusion of carbon atoms causes changes in material composition, material properties, and phase transformation kinetics. Secondly, temperature changes affect the nucleation and growth of phase and the temperature field, due to the generation of latent heat from the phase transformation. The stress–strain field changes due to the phase transition, which, in turn, induces expansion or contraction of the material. Conversely, the stress–strain can also inhibit or induce the formation of the phase transformation. Thirdly, due to the inevitable changes in the temperature field, expansion or contraction of the material, i.e., thermal strain, would occur. When large distortions occur within the material, as a result of processing and heat treatment, heat generation also occurs, leading to a change in the temperature field. It can be seen that the phenomenon of multi-field coupling should be considered in the carburizing and quenching simulation.

### 2.1. Theoretical Model of Non-Diffusion-Type Transformation (Martensite)

The martensitic transformation rate is related to the carbon concentration and the cooling rate, and the degree of transformation is higher when the degree of undercooling is greater. The K-M equation used for martensitic transformation can be described by Equation (1).

(1) $$\xi_{M}=1-\exp\left[-\alpha \left(M_{s}-T\right)\right]$$

In the formula, $\xi_{M}$ is the volume fraction of the martensite, *M_s_* is the initiation temperature of the martensite transformation, and *α* is a constant, usually taken as 0.011 in many calculations for most steels.

### 2.2. Theoretical Model of the Stress–Strain Field

The Thermal Prophet module based on the finite element method was utilized in the present simulations. The part distortion was predicted, taking into account the deformation based on the following strain components:

(2) $$d\varepsilon_{ij}=d\varepsilon_{ij}^{e}+d\varepsilon_{ij}^{p}+d\varepsilon_{ij}^{th}+d\varepsilon_{ij}^{tr}+d\varepsilon_{ij}^{tp}$$

where *e*, *p*, *th*, *tr*, and *tp* represent the contributions from elastic, plastic, thermal, phase transformation, and transformation plasticity, respectively. The strain due to transformation plasticity $d\varepsilon_{ij}^{tr}$ was considered in this calculation and can be described as Equation (3) [17,18].

(3) $$d\varepsilon_{ij}^{tp}=3K\sigma_{ij}\left(1-V\right)\cdot dV$$

where *K* is the transformation plasticity coefficient related to the microstructure, carbon content, and temperature of the steel; *V* is the volume fraction of the new phase structure; and *dV* is the volume formation rate of the new phase.

In order to obtain adequate simulation results, the material data should be accurate. The following information on materials properties have to be known for distortion prediction caused by quenching through numerical simulations [19]:

- Phase transformation kinetics, i.e., TTT and CCT diagrams.
- Temperature-dependent thermophysical properties for each phase formed, such as density, Young’s modulus, thermal expansion coefficient, and thermal conductivity.
- Temperature-dependent mechanical properties of each phase formed, including tensile strength, yield strength, and hardness.

The kinetics parameters of martensite transformation were identified by a thermal expansion experiment in the present work. The partial mechanical and thermophysical properties of the various materials and constituents (austenite, martensite, bainite, ferrite and pearlite) are taken into consideration and provided by JMatPro (Sente Sofeware Ltd., Surrey, UK) [20].

## 3. Methodology

### 3.1. Experimental Procedure of Heat Treatment

**Figure 2:**
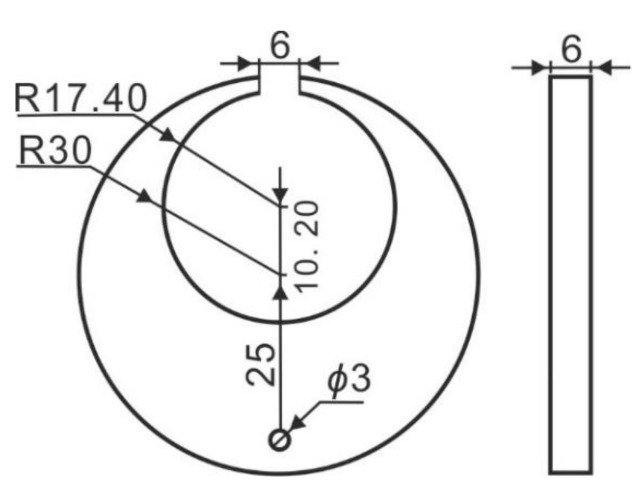
C-ring geometry (all dimensions in mm).

The material employed for the C-ring was a 20Cr2Ni4A steel, with a nominal composition of 0.17 wt.% C, 0.32 wt.% Si, 0.39 wt.% Mn, 1.35 wt.% Cr, and 3.57 wt.% Ni. The geometry and dimensions of the C-rings are given in Figure 2.

**Figure 3:**
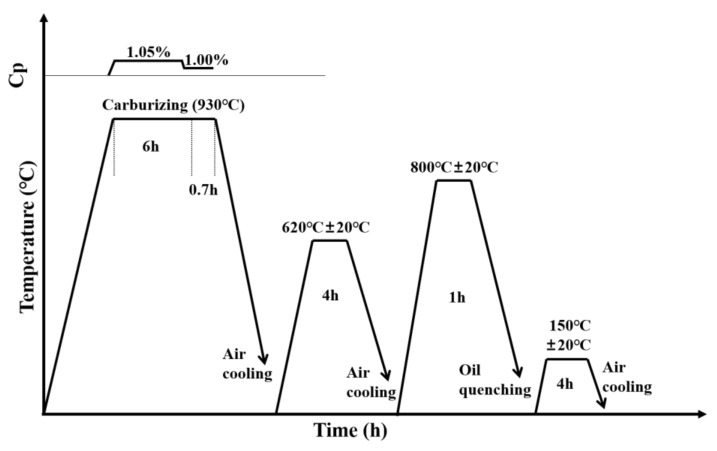
Preparation process of the C-Nary rings.

The C-rings were prepared via the process shown in Figure 3. The continuous gas carburizing temperature was 930 °C and the total time was 10 h in an Ipsen atmosphere furnace, followed by high-temperature tempering at 620 °C for 4 h. The samples were held at 800 °C for 1 h, then quenched through fast vertical movement of the ring (thickest part of the ring at the bottom and ring gap at the top) into the quenching oil (HQK) at 25 °C, where it was held for at least 300 s. After quenching, low-temperature tempering at 150 °C for 4 h was carried out. The carburizing and quenching process was simulated according to the actual process.

### 3.2. Phase Transformation Behavior Depending on Carbon Content

The carbon content of the 20Cr2Ni4A steel after carburizing changes significantly, which leads to changes in material parameters, such as the phase transformation kinetic parameters, elastic modulus, thermal expansion coefficient, and other parameters. The accuracy of these parameters plays a key role in the accuracy of the numerical simulation of the quenching heat treatment of the magic detective. Therefore, the thermal expansion experiment was carried out to obtain the transformation kinetics and thermal expansion coefficients of martensite and austenite under different carbon contents.

Five samples that differed only in carbon content were smelted by an electromagnetic induction furnace, and their chemical compositions, which were tested by an Inductively Coupled Plasma Emission Spectrometer iCAP 6300 Radial (Thermo Fisher Scientific, Walthamm, MA, USA), are shown in Table 1. The samples were processed for thermal expansion experiments with different cooling rates of 17.2, 8.6, 4.3, 1.72, 0.86, 0.28, 0.14, and 0.06 °C/s by using a thermal simulation testing machine Gleeble-3500 (Dynamic Systems Inc Corporation, New York, NY, USA).

### 3.3. Experimental Results of Martensitic Transformation Kinetics

**Figure 4:**
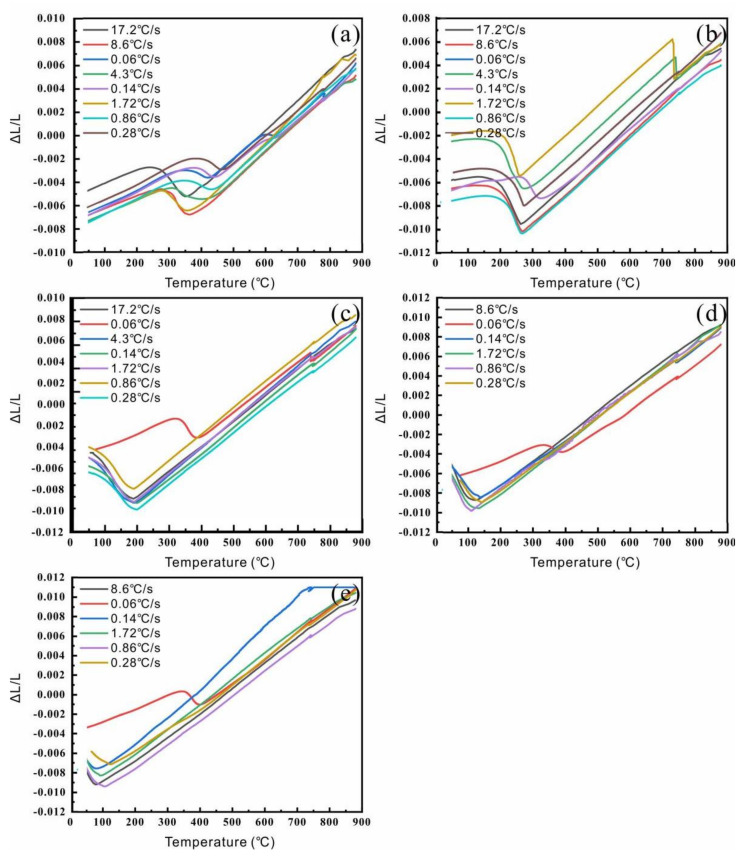
The thermal expansion curves with different samples at different cooling rates: (**a**) 20Cr; (**b**) 40Cr; (**c**) 60Cr; (**d**) 80Cr; (**e**) 100Cr.

**Figure 5:**
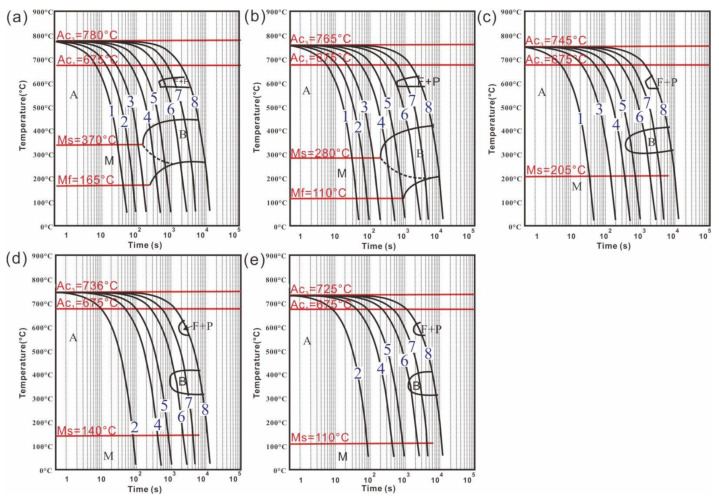
The CCT curves of samples with different carbon contents: (**a**) 20Cr; (**b**) 40Cr; (**c**) 60Cr; (**d**) 80Cr; (**e**) 100Cr (cooling rates: 1–17.2 °C/s, 2–8.6 °C/s, 3–4.3 °C/s, 4–1.72 °C/s, 5–0.86 °C/s, 6–0.28 °C/s, 7–0.14 °C/s and 8–0.06 °C/s; Abbreviation for Microstructure: A—Austenite, B—Bainite, F—Ferrite, P—Pearlite, M—Martensite).

The thermal expansion curves and CCT curves were obtained as shown in Figure 4 and Figure 5. According to the expansion curves, the phase transition point, phase expansion coefficient of the martensite and austenitic, and thermal expansion coefficient were obtained by the tangent method. The parameters of the phase transformation of the XXCr2Ni4A samples were obtained and are listed in Table 2. It can be seen that the austenitizing temperature Ac_3_, *M_s_* point, and thermal expansion coefficient of the martensite α_M_ decreased, while the phase expansion coefficient of the martensite $\beta_{M}^{0}$ increased with the increase in carbon content. The constant *α* in the K-M equation used for martensitic transformation is calculated based on Equation (1), and the results are greater than 0.011. The K-M equations for five kinds of carbon content are shown as Equations (4)–(8), which were used for the simulation.

In the formula, α is the linear expansion coefficient, L is the length after heat treatment, and L_0_ is the size before heat treatment.

(4) $$\xi_{M0.2\%}=1-\exp\;[-0.0288\left(M_{s}-T\right)]$$

(5) $$\xi_{M0.4\%}=1-\exp\;\left[-0.0271\left(M_{s}-T\right)\right]$$

(6) $$\xi_{M0.6\%}=1-\exp\;\left[-0.0295\left(M_{s}-T\right)\right]$$

(7) $$\xi_{M0.8\%}=1-\exp\;\left[-0.0281\left(M_{s}-T\right)\right]$$

(8) $$\xi_{M1.0\%}=1-\exp\;\left[-0.0249\left(M_{s}-T\right)\right]$$

where $\xi_{M\;x\%}$ is the volume fraction of the martensite (x = 0.2, 0.4, 0.6, 0.8, 1.0), *M_s_* is the initiation temperature of the martensite transformation, and *T* is the temperature

### 3.4. Experimental Results of Heat Transfer Coefficient

**Figure 6:**
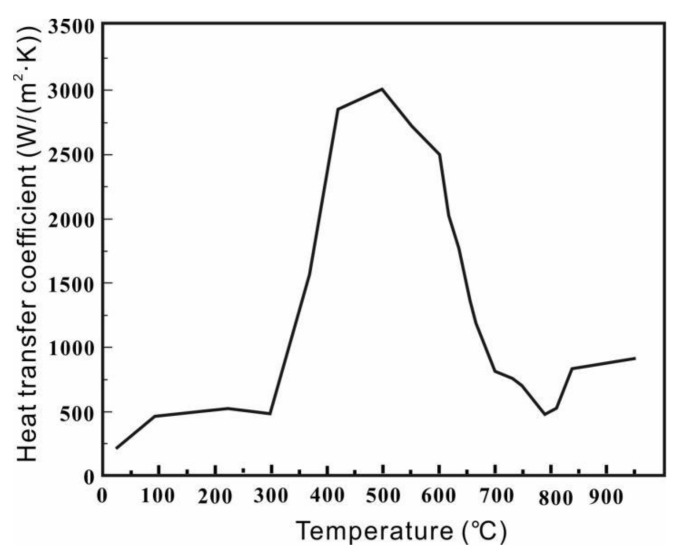
Heat transfer coefficient between 20Cr2Ni4A steel and HQG oil.

The quenching medium is the key factor affecting the degree of distortion and mechanical properties of the components during the quenching process. HQG rapid quenching oil was used for quenching. Its operating temperature is 60 °C and the maximum cooling rate reaches up to 90 °C/s. In order to improve the authenticity and accuracy of the quenching simulation results, the actual heat transfer coefficient is obtained by an inverse heat transfer calculation [21]. Figure 6 shows the variation in the heat transfer coefficient with temperature between 20Cr2Ni4A steel and HQG oil.

## 4. Results of Simulation

### 4.1. Time-Dependent Temperature and Phase Transformation

**Figure 7:**
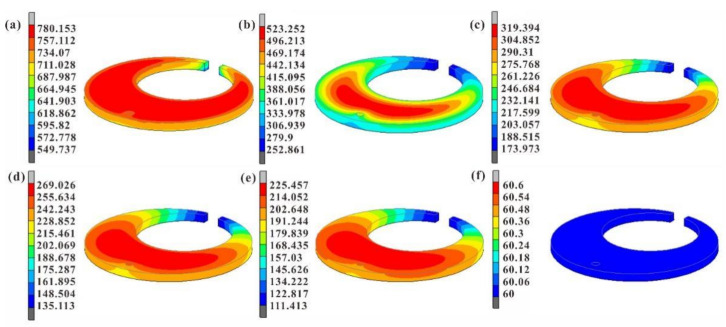
Temperature distribution of simulated predicted 20Cr2Ni4A steel C-type specimens with time: (**a**) 1 s, (**b**) 5 s, (**c**) 10 s, (**d**) 15 s, (**e**) 20 s and (**f**) 126 s.

In Figure 7, we can see the variation in and distribution of temperature clouds from 1, 5, 10, 15, 20, and 126 s with time during cooling of the C-ring specimen. As shown in Figure 7, the temperature at the notch of the C-ring cools the fastest, and the center part of the C-ring cools the slowest, due to the different thinness of the specimen and the different distance to the surface. From Figure 7b, it can be seen that after cooling for 5 s, the temperature of the notch position has been cooled to near 252 °C, but the center temperature of the specimen still reached 496 ℃. The temperature difference between the notch position and the center position of the specimen is large at this time, resulting in relatively large deformation. The temperature difference between the surface and core decreases with the increase in quenching time. When cooling to 126 s, the overall temperature of the C-ring is 60 °C, which is the same as the oil.

**Figure 8:**
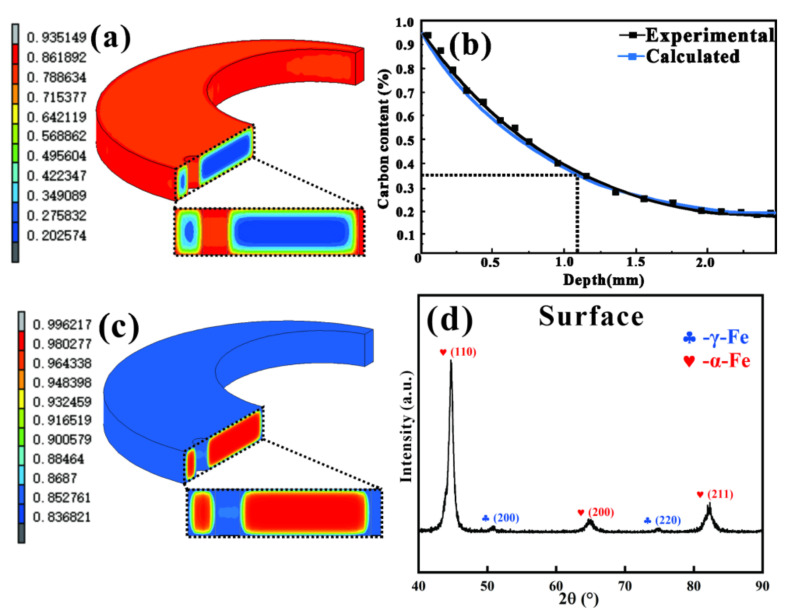
(**a**) Carbon concentration distribution; (**b**) the calculated and experimental carbon concertation distribution of the carburized layer; (**c**) martensite content distribution at different positions; (**d**) XRD diffraction pattern measured by the experiment on the surface layer of the specimen.

Figure 8a shows the cross-sectional distribution of carbon concentration of the C-ring of 20Cr2Ni4A steel after gas carburizing at 930 °C and quenching at 800 °C. It can be seen that the carbon concentration on the surface of the specimen can reach up to 0.93%, and from the local enlargement of the cross-section, it can be seen that the carbon concentration was 0.42~0.34%, showing a slow gradient trend; finally, the carbon concentration in the core was 0.20%. The carbon concentration profiles of the actual samples were also measured using a glow discharge photoemission spectrometer-LECO GDS850A. As shown in Figure 8b, the actual measured carbon concentration profile has good agreement with the simulated values. Figure 8c shows the simulated evolution of the martensite volume fraction of the C-ring after heat treatment, from which it can be seen that less martensite was formed on the surface after quenching due to the increase in surface carbon content after carburizing, leading to a decrease in the surface martensite *M_s_* point from 370 to 140 °C. The simulated residual austenite content of the surface was 16.32%. The XRD diffraction method was used to determine the retained austenite content, and its XRD diffraction pattern of the surface is shown in Figure 8d. Based on Equation (9), the retained austenite content was calculated to be 18.75%, which is a little higher than 14% in ref [22] with a slightly lower carbon content on the surface. The experimental results in the present work fit well with the simulation.

(9) $$V_{\gamma }=\frac{1.4I_{\gamma }}{I_{\alpha }+1.4I_{\gamma }}$$

where *Vγ* is the volume fraction of retained austenite; *Iγ* is the mean integrated intensities of the austenite peaks, including *γ*(111), *γ*(200) and *γ*(220); and Iα is the mean integrated intensities of the martensite peaks, including *M*/*α*(110), *M*/*α*(200) and *M*/*α*(211).

**Figure 9:**
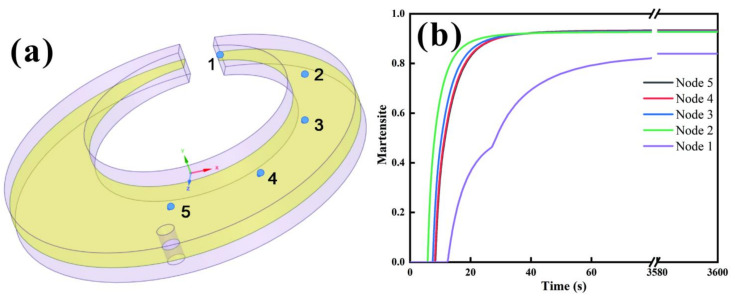
(**a**) Schematic diagram of simulated sites; (**b**) martensite volume versus time graph.

As shown in Figure 9a, five different typical locations from the notched end to the core of the C-ring were selected in turn, marked as Node 1, Node 2, Node 3, Node 4 and Node 5, where Node 1 is the outer surface. The variation in martensite content with time at each point during quenching was simulated, as shown in Figure 9b. It can be seen that the martensitic transformation at point 1 occurred at the beginning of 12.53 s, which was more consistent with the temperature field shown in Figure 7c,d. At this time, the carbon concentration ranged from 0.788 to 0.935 wt.%, and the corresponding M_s_ temperature was 140–110 °C. This indicates that the simulation accuracy of the relationship between carbon concentration, temperature field, and martensite content made by 20Cr2Ni4A steel is better for C-rings.

**Figure 10:**
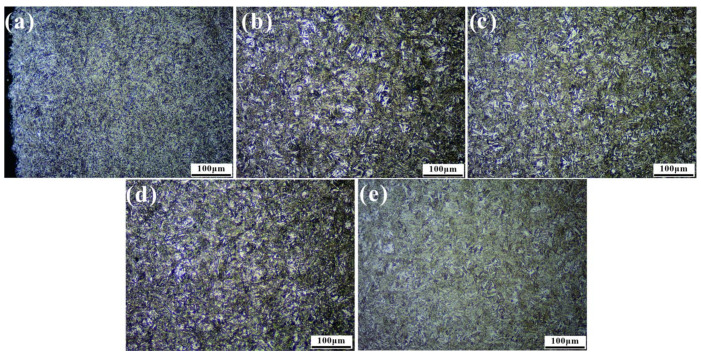
OM microstructure of 20Cr2Ni4A steel C-ring specimens at different locations: (**a**) Node 1, (**b**) Node 2, (**c**) Node 3, (**d**) Node 4 and (**e**) Node 5.

The microstructure of the C-ring at different points is shown in Figure 10. As can be seen from Figure 10a, the surface layer has typical carburized layer organization, consisting of black high-carbon needle-like martensite, white-bright residual austenite blocks, and carbides. The matrix is all low-carbon slate-like martensite, with slight differences in the size of the martensite slat bundles depending on the temperature field, but no changes in the tissue composition were found. This indicates that the actual tissue composition was consistent with the simulated martensite content.

### 4.2. Distortion and Displacement Distribution

**Figure 11:**
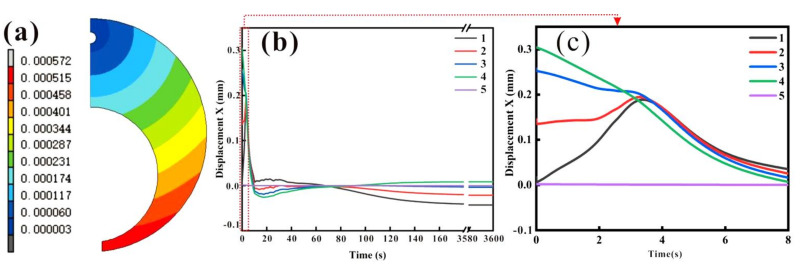
Simulated quenching deformation (**a**) in the x direction of the points in the longitudinal cross-section of the C-ring (displacement magnified 20×); (**b**) with time at typical position of C-Nary rings; (**c**) enlarged view of the position marked with dotted line in the (**b**).

**Figure 12:**
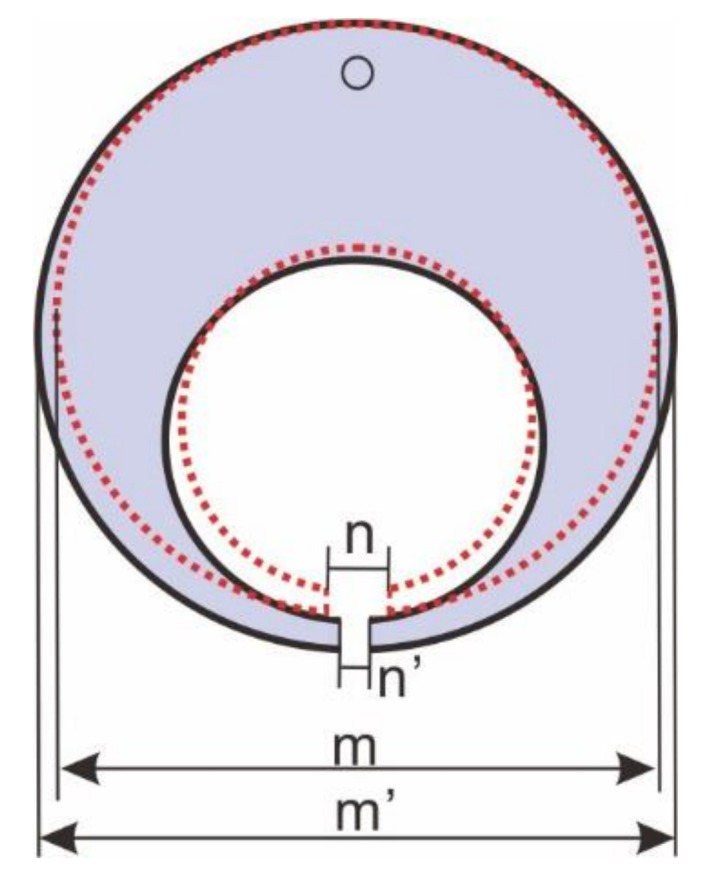
C-ring specimen before heat treatment (m and n dimensions) and after heat treatment (m’ and n’ dimensions).

The distortion after carburizing and quenching is an important component of a quality check. If the amount of transformation is too large, it will lead to gear transmission unstable and produce a large noise level, which can affect the assembly of the transmission [12]. Figure 11a is a 20-fold magnification of the final state deformation of the C-ring after carburizing and quenching. Figure 11b displays the simulated quenching deformation with time at a typical position of the carburized C-ring for a period of 100 s after quenching. The measurements of the ring dimensions before and after quenching were performed in an ATOS Core 135 with an accuracy of 0.01 mm. Two dimensional changes were analyzed: gap opening (*G*) and outside diameter (*OD*). Based on Figure 12, the dimensional changes may be expressed as follows [6]:

*G* = *n*’ − *n* (10)

*OD* = *m*’ − *m* (11)

The ring gap closed 0.129 mm and the outer diameter expanded about 0.032 mm. As shown in Table 3, the relative differences between gap opening and outside diameter increase were smaller than 8%. The calculated distortions of the gap and the outer diameter were in good agreement with the measured value. The amount of expansion was also different at the start of quenching for the five positions, while the deformation of each position showed different changing laws as the quenching time increased.

**Figure 13:**
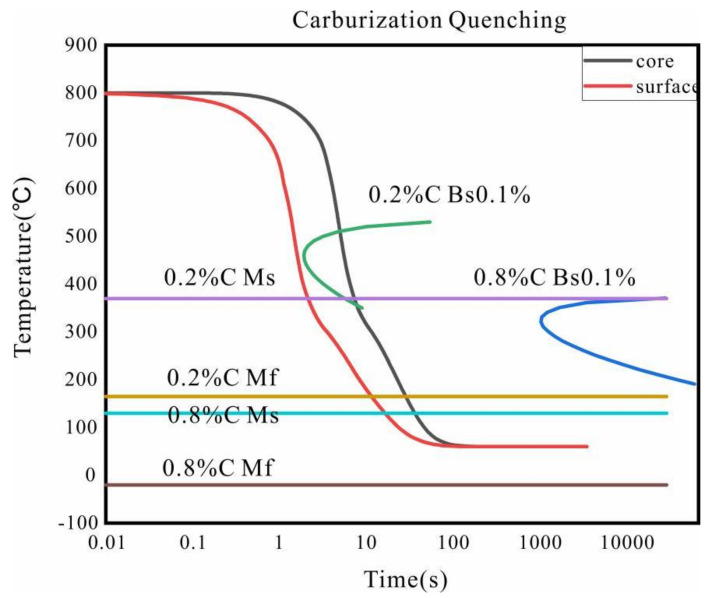
Schematic diagram of the cooling phase transformation process of the surface and core of the carburized sample.

A large number of studies have shown that deformation from carburizing and quenching is mainly caused by the unsynchronized and uneven martensitic transformation stress between the carburized layer and the core matrix. The martensitic transformation stress is closely related to the transformation process. Figure 13 shows the schematic diagram of the phase transformation process of the carburized layer (surface) and the center (Center) of the carburized sample, from which we can understand the deformation law of carburizing and quenching. Due to the big difference in carbon content between the core (only about 0.20%) and the carburized layer (the maximum value), the martensitic transformation temperature *M_s_* changed from 370 to 140 °C, and the end temperature of the martensitic transformation *M_f_* decreased from 165 to −20 °C, as shown in Figure 13. As we can see, the *M_f_* of the 0.8 wt.% carbon content sample was lower than room temperature, leading to an increase in the retained austenite content. In addition, there was a temperature difference between the core and the surface. The core underwent martensitic transformation and expanded at the initial stage of quenching, while the carburized layer was still in the austenite state. In addition, the volume expansion due to the phase transformation of the surface with high carbon content was larger than that of the core, which eventually led to the formation of stress. The surface of the C-ring was under compressive stress, and exhibited shrinkage deformation along the diameter direction after quenching.

In summary, the model simulated the gear steel after carburizing and quenching, considering the multi-field coupling effect of temperature field, stress–strain field, and phase transformation field. The distributions of carbon concentration, microstructure, and deformation were predicted, which were in good agreement with the experimental results. This indicates that the thermophysical parameters used were accurate and the model could be used as a guide for practical application. These provide a prerequisite for the subsequent realization of the microstructure, as well as performance optimization and micro deformation control of gear steel.

## 5. Conclusions

The carburizing and quenching process of the 20Cr2Ni4A steel was simulated by using a finite element simulation. Based on the simulation and experimental results, we obtained the following conclusions:

(1) The phase transformation parameters, depending on the carbon content and heat transfer coefficient between the steel and HQG oil, were obtained to improve the accuracy of the carburizing and quenching simulation.
(2) The distortion and microstructure of the C-ring after carburizing and quenching were predicted by considering the effect of phase transformation strain. The measured results concerning distortion and microstructure were in good agreement with the simulated values.
(3) The methodology used to predict the distortion during carburizing and quenching may be applied to parts of various shapes and materials. Therefore, the heat treatment process may be included in the process design to obtain the final product dimension.

## Figures and Tables

**Table 1:** The composition of the XXCr2Ni4Asteel (wt.%).

	C	Si	Mn	Cr	Ni
20Cr	0.18	0.31	0.52	1.46	3.31
40Cr	0.38	0.33	0.51	1.38	3.44
60Cr	0.57	0.35	0.52	1.37	3.43
80Cr	0.77	0.36	0.51	1.35	3.42
100Cr	1.02	0.36	0.52	1.34	3.40

**Table 2:** The parameters obtained from the expansion experiment.

	20Cr	40Cr	60Cr	80Cr	100Cr
Ac_3_/°C	780	765	745	736	725
*M_s_*/°C	370	280	205	140	110
*α_A_*	2.402 × 10^5^	2.456 × 10^5^	2.320 × 10^5^	2.494 × 10^5^	2.319 × 10^5^
*α_M_*	1.242 × 10^5^	1.143 × 10^5^	1.044 × 10^5^	0.945 × 10^5^	0.846 × 10^5^
$\beta_{M}^{0}$	0.008631	0.009824	0.01107	0.012210	0.013403
*α*	0.0288	0.0271	0.0295	0.0281	0.0249

**Table 3:** Comparison of the experimental and simulated results for the geometric distortions of the C-ring.

Position	Experimental Result	Simulation Result	Prediction Difference
Gap opening (mm)	−0.14	−0.129	7.8%
Outside diameter (mm)	0.03	0.032	6.25%

## Data Availability

The raw/processed data required to reproduce these findings cannot be shared at this time as the data also forms part of an ongoing study.
